# Mid-dose losartan mitigates diabetes-induced hepatic damage by regulating iNOS, eNOS, VEGF, and NF-κB expressions

**DOI:** 10.3906/sag-1901-15

**Published:** 2019-10-24

**Authors:** Fatih OLTULU, Aylin BUHUR, Çevik GÜREL, Gökçe Ceren KUŞÇU, Melih DAĞDEVİREN, Nefise Ülkü KARABAY YAVAŞOĞLU, Timur KÖSE, Altuğ YAVAŞOĞLU

**Affiliations:** 1 Department of Histology and Embryology, Faculty of Medicine, Ege University, İzmir Turkey; 2 Department of Histology and Embryology, Faculty of Medicine, Harran University, Şanlıurfa Turkey; 3 Department of Biology, Faculty of Science, Ege University, İzmir Turkey; 4 Department of Biostatistics and Medical Informatics, Faculty of Medicine, Ege University, İzmir Turkey

**Keywords:** Hepatic damage, angiotensin II receptor blocker, iNOS, eNOS, VEGF, NF-κB

## Abstract

**Background/aim:**

Losartan, an antihypertensive drug, is highly preferred in patients with diabetes mellitus (DM) and hypertension because of its retarding effect on diabetic nephropathy. In this study, we investigated the potential therapeutic effect of different doses of losartan on hepatic damage in a streptozotocin (STZ, 50 mg/kg)-induced DM model in rats.

**Materials and methods:**

In this study, five different groups were formed: control, DM, low-dose losartan (5 mg/kg), mid-dose losartan (20 mg/kg), and high-dose losartan (80 mg/kg). Liver tissues of experimental groups were evaluated immunohistochemically for TUNEL, iNOS, eNOS, VEGF, and NF-κB pathways. In addition to immunohistochemical analysis, analyses of SOD and MDA, which are oxidative stress markers, were also performed and the results were evaluated together.

**Results:**

When biochemical and immunohistochemical findings were evaluated together, it was found that the results obtained from the mid-dose losartan group were closer to those of the control than the other groups.

**Conclusion:**

This study indicated that mid-dose losartan administration may have a therapeutic effect by inhibiting apoptosis and regulating iNOS, eNOS, VEGF, and NF-κB protein expressions in DM-induced hepatic damage.

## 1. Introduction

Diabetes mellitus (DM) is a chronic metabolic disorder characterized by increased blood glucose level, hyperglycemia, and glucosuria, which are usually caused by deficiency in insulin secretion and/or insulin hormone activity in the pancreatic beta cells [1,2]. Chronic hyperglycemia arising from untreated DM can cause damage to several organs such as the eyes, kidneys, heart, and liver [3]. For example, many studies have shown that DM may be a trigger in different liver diseases such as nonalcoholic fatty liver (NAFLD), nonalcoholic steatohepatitis, fibrosis, cirrhosis, and later hepatocellular carcinoma (HCC) [4].

Nitric oxide (NO) is an important signaling molecule produced by three different nitric oxide synthase (NOS) isoforms: neuronal NOS (nNOS; NOS1), inducible NOS (iNOS; NOS2) and endothelial NOS [5]. Nitric oxide derivatives are involved in biological processes such as immune activation, cellular communication, metabolism, and blood pressure regulation and play a paradoxical role in the regulation of liver physiology and pathophysiology [6,7]. For example, NO expressed by eNOS in the liver sinusoidal endothelial cells (LSECs), portal vein, central vein, and lymphatic vessels protects the homeostasis of the liver and inhibits the formation of pathological conditions in the liver [8]. In contrast, NO expressed by iNOS in various liver cells, including LSECs, hepatocytes, and Kupffer cells (hepatic stellate cells), plays a role in the emergence of liver pathologies by triggering the formation of reactive nitrogen species (RNS) [9]. In addition, studies have shown that hyperglycemia activates iNOS expression, which can cause inflammation, apoptosis, and other diabetic complications in the liver [10]. On the other hand, oxidative stress induced by hyperglycemia causes an increase in the expression of vascular endothelial growth factor (VEGF), which is considered to be the key regulator of both physiological and pathological angiogenesis [11]. It has been demonstrated by studies that VEGF has a role in the pathogenesis of diabetic complications by inducing NOS expressions, resulting in an increase in NO production [12]. It is also known that VEGF has a fibrinogenic effect on the liver via the triggering of inflammation and the stimulation of endothelial and stellate cells [13].

Nuclear factor κB (NF-κB), an important factor in the regulation of processes such as inflammation and apoptosis, is another molecule that changes the activation pattern in DM. Activation of NF-κB due to hyperglycemia caused by DM induces the expression of RNS and reactive nitrogen species (ROS) directly or indirectly and paves the way for DM-induced hepatic damage. It has been suggested that inappropriate activation of NF-κB may lead to hepatocellular carcinomas by increasing insulin resistance in diabetic mice and may increase the likelihood of hepatic steatosis in cirrhosis, thus causing more hepatic damage [14,15]. In addition, increased ROS due to hyperglycemia activates many different gene expressions, including iNOS, via the NF-κB pathway [16]. 

Losartan, an angiotensin II type I receptor antagonist, is an antihypertensive drug that has a delaying effect on diabetic nephropathy, especially in patients with DM and hypertension [17]. On the other hand, in a study conducted with chronic hepatitis C patients in 2005, it was found that 6-week losartan treatment reduced subendothelial fibrosis in the liver [18]. 

In this study, we investigated the therapeutic effect of different doses of losartan on hepatic damage in a streptozotocin (STZ)-induced diabetic rat model. The liver tissues of the experimental groups were evaluated immunohistochemically for iNOS, eNOS, VEGF, and NF-κB pathways. 

## 2. Materials and methods

### 2.1. Animals

Thirty-five adult male Wistar albino rats (weighing between 200 and 250 g) were used in this study. Rats caged in controlled rooms with 22 ± 3 °C temperature and 12-h light/dark cycles were fed with standard rat feed and water ad libitum. Experimental procedures used in this study were approved by the Ege University Local Ethics Committee for Animal Experiments. All procedures were carried out in strict compliance with the animal experiment guidelines prepared for the care and use of laboratory animals.

### 2.2. Experimental design

DM was induced in 28 rats by intraperitoneal injection of STZ (Sigma-Aldrich, St. Louis, MO, USA) (50 mg/kg in 0.1 M citrate buffer, pH 4.5). No drug was administered to the other 7 rats that all had blood glucose levels of <120 mg/dL (control group, n = 7). DM was verified after 24 h by evaluating the blood glucose levels. Rats with blood glucose levels of >250 mg/dL were included in the study as the diabetic rat group (n = 28) [19]. The 28 diabetic rats were then randomly separated into 4 groups. For a period of 4 weeks, the DM group (n = 7) was given no medication. The DM + low-dose losartan group (n = 7) was administered 5 mg/kg/day oral losartan (Cozaar, 50 mg; Merck Sharp & Dohme, Kenilworth, NJ, USA) for a 4-week period. The DM + mid-dose losartan group (n = 7) was administered 20 mg/kg/day oral losartan for a 4-week period. The DM + high-dose losartan group (n = 7) was administered 80 mg/kg/day oral losartan for a 4-week period. 

At the end of the study, blood samples of 1 mL were taken into heparinized tubes for biochemical analysis from the rats, which were sedated under anesthesia. After bloodletting, liver tissues of euthanized rats were rapidly dissected for histopathological and immunohistochemical examinations.

### 2.3. Analysis of lipid peroxidation 

Blood samples collected by cardiac puncture under sterile conditions were centrifuged at 4 °C and 1000 × *g* for 15 min so that blood plasmas were obtained. Plasma samples frozen rapidly on dry ice were stored at –80 °C until they were used. Lipid peroxidation was determined by measuring malondialdehyde (MDA) levels in plasma samples. In this respect, MDA levels were determined in accordance with the instructions of a commercially available lipid peroxidation (MDA) colorimetric/fluorometric assay kit (BioVision, Milpitas, CA, USA). The absorbance of each sample was measured at 532 nm with an ELISA plate reader (PolarSTAR Omega, BMG LABTECH, Germany) and results were obtained.

### 2.4. Analysis of serum superoxide dismutase (SOD) activity

Blood samples collected by cardiac puncture under sterile conditions were centrifuged at 4 °C and 1000 × g for 15 min so that blood plasmas were obtained. Plasma samples frozen rapidly on dry ice were stored at –80 °C until they were used. The SOD levels of the groups were determined according to a commercially available SOD activity assay kit (BioVision). The absorbance of each sample was measured at 450 nm with an ELISA plate reader (PolarSTAR Omega, BMG LABTECH) and results were obtained. 

### 2.5. Histological analysis of liver tissues

The rats were euthanized under combined ketamine (60 mg/kg, Ege Vet, Alfamine, Alfasan International B.V., Woerden, the Netherlands) and xylazine (10 mg/kg, Ege Vet, Alfazyne, Alfasan International B.V.) anesthesia. Liver tissues were fixed for 48 h in 4% paraformaldehyde. Afterwards, 5-µm sections were taken from the tissues embedded in paraffin blocks using routine protocols. Sections were stained with hematoxylin and eosin (H&E) after deparaffinization and dehydration. The tissues were photographed with a digital camera (C-5050, Olympus, Tokyo, Japan) mounted on a microscope (BX5, Olympus) after staining.

### 2.6. Immunoexpressions of iNOS, eNOS, VEGF, and NF-κB

Sections were incubated with 10% H2O2 (Sigma-Aldrich) for 10 min for endogenous peroxidase blockade. To prevent nonspecific antibody–antigen binding, sections were incubated with Super Block (ScyTec Inc., Greenwood Village, CO, USA) for 1 h at room temperature and washed with PBS. After this step, sections were incubated with 1:200 diluted primary antibodies (iNOS, eNOS, VEGF, and NF-κB, Santa Cruz Biotechnology, Santa Cruz, CA, USA) for 24 h at 4 °C. At the end of this time, the sections were respectively incubated with biotinylated secondary antibody (ScyTec Inc.) and horseradish peroxidase conjugated streptavidin (ScyTec Inc.). Finally, the contrast staining of the sections incubated with diaminobenzidine (DAB) was performed with Mayer’s hematoxylin (Merck, Germany). Sections were cleaned with xylol and then closed with Entellan (Merck) [20].

### 2.7. Analysis of terminal-deoxynucleotidyl transferase dUTP nick end labeling (TUNEL) 

TUNEL analysis was performed to determine apoptosis in liver tissues belonging to groups. After TUNEL staining was performed according to the procedure of the ApopTag Peroxidase In Situ Apoptosis Detection Kit (Merck), the sections were photographed and the percentages of TUNEL-positive cells between all experimental groups were compared.

### 2.8. Statistical analysis

Data analysis was performed with SPSS 15.0 for Windows (SPSS Inc., Chicago, IL, USA). Comparisons were then made between the control and treatment groups using one-way analysis of variance followed by a Tukey post hoc test. Values were presented with mean standard errors and P < 0.05 was considered statistically significant.

## 3. Results

### 3.1. SOD and MDA findings

**Figure 1:**
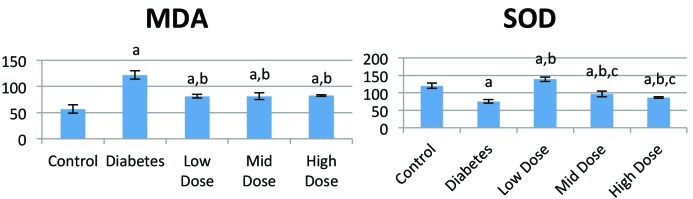
MDA levels (nmol/mL) and SOD activities (% inhibition rate) of rat blood plasmas. a: Statistically significant compared to control group (P < 0.05), b: statistically significant compared to diabetes group (P < 0.05), c: statistically significant compared to high-dose losartan group (P < 0.05).

In the SOD analysis it was found that SOD activation was higher in the losartan-treated groups than in the DM group (P < 0.05). On the other hand, MDA levels were significantly lower in losartan-treated groups than in the diabetic group (P < 0.05) (Figure 1). 

### 3.2. Histological findings

There were regular hepatocytes extending radially around the central vein in the sections of the control group. Sinusoids and Kupffer cells were normal and there were no signs of hemorrhage and infiltration in this group.

The number of hepatocytes in the DM group decreased and there were losses in the radial configuration of hepatocytes. Hepatocyte deformation and vacuolization were noted. Hemorrhage and infiltration were present. 

**Figure 2:**
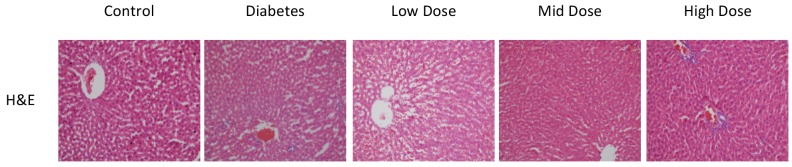
H&E staining of sections from all experimental groups. Control group livers showed normal radially extending hepatocytes. DM group showed decreased radial configuration of hepatocytes and vacuolation. Low-dose losartan group showed little improvement in contrast with diabetes group. Mid-dose losartan group showed similar configuration to control group. High-dose losartan showed increased number of Kupffer cells and infiltration compared to other losartan-treated groups (20× magnification).

The findings for the mid-dose losartan group were found to be closer to those of the control group compared to other groups. In the high-dose group, there was no difference in the number of hepatocytes and their regulation and sinusoids compared to the medium dose, whereas an increase in the number of Kupffer cells and infiltration were noted (Figure 2).

### 3.3. Immunohistochemical findings

In histochemical analyses, it was found that iNOS protein expression increased and eNOS protein expression decreased in the DM group compared to the control and losartan-treated groups (P < 0.05). However, a level of iNOS protein expression close to that of the control group was determined in the mid-dose losartan group, and in the group with high-dose losartan, an increase in iNOS expression was observed. 

We observed increased VEGF protein expression in the livers of diabetic rats (P < 0.05). It is noteworthy that in groups treated with losartan, there was a decrease in VEGF protein expression, just as in iNOS expressions. We also observed that VEGF expression was more similar to the control group in the mid-dose losartan treatment group compared to the other groups. 

**Figure 3:**
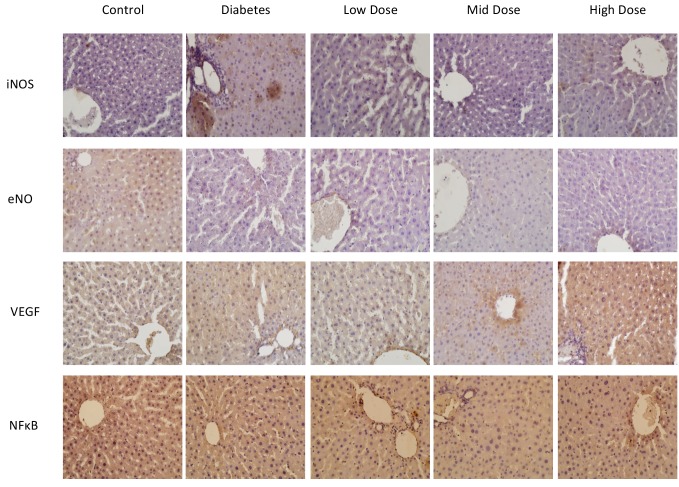
iNOS, eNOS, VEGF, and NF-κB immunostaining of sections from all groups (40× magnification).

NF-κB immunoexpression levels were observed to be higher in the DM group than in the control group. When losartan-treated groups were evaluated among themselves, it was found that NF-κB expression was significantly lower in the mid-dose losartan group compared to the other groups (P < 0.05) (Figure 3). The immunoexpression levels and the P-values of the groups are shown in the Table.

**Table:** Immunoexpression levels and TUNEL scores of control and other experimental groups

	Control	DM	Low-dose	Mid-dose	High-dose	P value
Immunoexpression levels						
iNOS	1 ± 0.258	3 ± 0.378	2 ± 0.32	1,5 ± 0.32	2 ± 0.258	0.000
eNOS	3 ± 0.378	1 ± 0.258	1.5 ± 0.353	2 ± 0.378	1.5 ± 0.176	0.000
VEGF	1 ± 0.231	3 ± 0.231	1.5 ± 0.258	2.5 ± 0.258	3 ± 0.378	0.000
NF-ᴋB	1 ± 0.231	2.75 ± 0.267	2.5 ± 0.258	1.5 ± 0.231	2 ± 0.231	0.000
						
TUNEL scores (%)						
TUNEL-positive cells	2 ± 0.175%	4 ± 0.375%	3 ± 0.312%	2 ± 0.212%	3 ± 0.262%	0.000

Groups	SOD activation(% inhibition rate)	MDA levels(nmol/mL)
Control	120.15 ± 7.39	56.98 ± 8.22
DM	75.37 ± 5.28a	122.09 ± 8.22a
Low-dose	138.81 ± 6.33a,b	81.4 ± 3.29a,b
Mid-dose	97.01 ± 8.44a,b,c	81.4 ± 6.58a,b
High-dose	86.57 ± 2.11a,b	82.56 ± 1.64a,b

### 3.4. TUNEL staining findings

**Figure 4:**
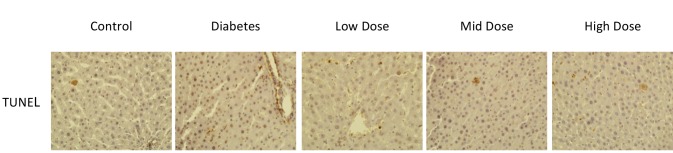
TUNEL staining of sections from control and other groups (40× magnification).

In TUNEL staining analysis, there was an increase in the number of apoptotic cells in the diabetic group compared to the control group. In addition, when the diabetic groups were evaluated among themselves, the number of TUNEL-positive cells decreased in the mid-dose losartan group compared to the other groups (P < 0.05) (Figure 4). TUNEL scores and the P-values of the groups are shown in the Table.

## 4. Discussion

DM is a common metabolic disorder characterized by hyperglycemia, which occurs due to impaired insulin secretion in the pancreas [21]. This disease, which causes disruptions in the metabolism of carbohydrate, lipids, and proteins in the body, prepares the ground for serious functional and structural pathologies in many organs such as the heart, kidneys, eyes, and liver in the long term [22]. For example, previous studies suggested that DM is associated with liver diseases such as cirrhosis, NAFLD, fibrosis, and HCC [23]. The main mechanisms involved in the development of DM-induced liver diseases are ROS production and oxidative stress induced by hyperglycemia [16]. ROS production and oxidative stress induced by hyperglycemia enhance NO formation in diabetic liver damage. Interacting with ROS, NO induces many cellular signaling pathways, causing lipid peroxidation and protein nitration and thereby leading to DM-induced tissue damage [24,25]. NO, which has a significant role in diabetic liver damage, is produced by three different NOS isoforms: neuronal NOS (nNOS; NOS1), inducible NOS (iNOS; NOS2), and endothelial NOS (eNOS; NOS3) [6]. The role of NO in liver physiology is paradoxical [26]. For example, NO expressed by eNOS in the LSECs, portal vein, central vein, and lymphatic vessels protect hepatocytes and endothelial cells against ischemic reperfusion injury [27]. On the other hand, it has been found that NOS produced by iNOS might exacerbate liver damage after the inflammatory response [28]. In addition, Ingaramo et al. showed that iNOS expression and NO level increased in diabetic liver injury and this increase could cause significant impairment of liver function [29]. Jeddi et al. found a decrease in eNOS expression and an increase in iNOS expression in the hearts of diabetic rats. They argued that this might play an important role in the pathophysiology of DM-associated cardiac problems [30]. In our study, increased iNOS protein expression and decreased eNOS protein expression were determined in the livers of diabetic rats. However, the level of iNOS protein expression closest to the control group was determined in the mid-dose losartan group. Studies on renal fibrogenesis [31] and left ventricular remodeling [32] by different groups have shown that mid-dose losartan suppresses iNOS mRNA and protein expression. In addition, Matsuhisa et al. reported that losartan triggered iNOS-to-eNOS dependence [32]. When the findings of this study are evaluated together with the literature, the present study clearly suggests that mid-dose losartan may be a candidate for modulation of NOS derivatives that play different roles in liver injury. 

Hyperglycemia-induced oxidative stress causes an increase in the expression of some enzymes and growth factors [33]. For example, VEGF, which is considered to be the key regulator of both physiological and pathological angiogenesis, is one of the growth factors that have increased expression in hyperglycemia-induced oxidative stress [11]. Also, studies have shown that VEGF, which is known to cause an increase in NO production by inducing eNOS and iNOS mRNA expressions, plays a role in the pathogenesis of diabetic complications and especially in retinopathy [12]. In their study of diabetic retinopathy, Djordjevic et al. observed an increase in the concentration of VEGF, which is thought to be very important for the destruction of the blood–retinal barrier and for neovascularization, with an increase in oxidative damage markers and iNOS concentration [33]. In another study Liu et al. showed increased expression of iNOS and VEGF in the cochlea of diabetic rats [34]. In our study, we observed increased expressions of iNOS and VEGF protein in the livers of diabetic rats. However, in groups treated with losartan, there was a decrease in VEGF protein expression, just like the iNOS expressions. In addition, we found that VEGF expression was more similar to the control group in the mid-dose losartan treatment group. Similar to our findings in this study, there is evidence that losartan suppresses VEGF mRNA and protein expression in different studies [35,36]. Also, Kamper et al. suggested that losartan administration in diabetic rats suppresses the production of VEGF and excess NO in the pancreas; thus, losartan can exert an antioxidant effect by suppressing oxidative and nitrosative stress [37].

NF-κB is a transcription factor that is required for the expression of most proinflammatory molecules, including enzymes, cytokines, and chemokines [38]. In vitro and in vivo experiments show that NF-κB activation plays a key role in the pathobiology of DM [39]. In this context, studies have shown that increased ROS levels associated with DM cause NF-κB activation, while this activation induces transcription of inflammatory genes such as cyclooxygenase-2 (COX-2), tumor necrosis factor-alpha (TNF-α), interleukin 1 (IL-1), and iNOS [40]. In addition to these studies, Pan et al. showed that the activation of NF-κB in diabetic cardiomyocyte cells leads to apoptosis, and inhibition of this pathway corrects cardiac dysfunction in diabetic mice [41]. These studies showed that NF-κB has an important role in DM-induced apoptosis. In our study, we determined that NF-κB immunoexpression intensity was higher in the diabetic groups than in the control group. When losartan-treated groups were evaluated among themselves, it was found that NF-κB expression was lower in the mid-dose group than the other groups. Also, our TUNEL staining analysis showed that there was an increase in the number of apoptotic cells (TUNEL-positive cells) in the diabetic group compared to the control group. In addition, when the diabetic groups were evaluated among themselves, the number of TUNEL-positive cells was decreased in the mid-dose group compared to the other groups. A number of past and present studies suggest that lipid peroxidation is one of the major causes of apoptotic injury associated with DM [42]. For example, Oyenihi et al. found increases in lipid peroxidation products such as MDA in the livers of diabetic rats but a decrease in the activity of antioxidant enzymes such as SOD [43]. The same group also revealed that TUNEL-positive cells were increased in diabetic rats, correlated with lipid peroxidation [43]. However, there are reports that losartan protects podocytes from apoptosis [44], cleans lipid peroxidation products in retinal [45] and pancreatic cells [37], and inhibits NF-κB activation [46]. The results of the present study support these reports.

In conclusion, these results suggested that mid-dose losartan administration may have a therapeutic effect by inhibiting apoptosis and regulating iNOS, eNOS, VEGF, and NF-κB protein expressions, which have been shown to play a role in the pathogenesis of DM-induced hepatic damage. Investigating the effects of the drugs such as losartan used in clinics for many years on the comorbidities observed in diabetic individuals may be effective in improving the quality of life of diabetic individuals and the emergence of new approaches in the treatment of this disease.

## Acknowledgement/Disclaimers/Conflict of interest

This study was supported by the Ege University Scientific Research Projects Coordination Unit [grant number: 17-ILAM-002 (to Altuğ Yavaşoğlu)]. The experimental procedures on the animals in this study were performed at Ege University, Drug Research and Development and Pharmacokinetic Applications (ARGEFAR).
